# Water dynamics in MCF-7 breast cancer cells: a neutron scattering descriptive study

**DOI:** 10.1038/s41598-019-45056-8

**Published:** 2019-06-18

**Authors:** Murillo L. Martins, Alexander B. Dinitzen, Eugene Mamontov, Svemir Rudić, José E. M. Pereira, Rasmus Hartmann-Petersen, Kenneth W. Herwig, Heloisa N. Bordallo

**Affiliations:** 1 Niels Bohr Institute, University of Copenhagen, DK-2100 Copenhagen, Denmark; 2 System and Production Engineering Graduate Program, Pontifical Catholic University of Goias, 74605-010 Goiania, Brazil; 3 Department of Biology, University of Copenhagen, DK-2200 Copenhagen, Denmark; 4 Neutron Scattering Division, Neutron Sciences Directorate, Oak Ridge National Laboratory, Oak Ridge, TN 37831 United States; 5 ISIS Facility, Rutherford Appleton Laboratory, Chilton, Didcot OX11 OQX UK; 6 European Spallation Source, PO Box 176, SE-221 00 Lund, Sweden

**Keywords:** Biomaterials - cells, Soft materials

## Abstract

Water mobility in cancer cells could be a powerful parameter to predict the progression or remission of tumors. In the present descriptive work, new insight into this concept was achieved by combining neutron scattering and thermal analyses. The results provide the first step to untangle the role played by water dynamics in breast cancer cells (MCF-7) after treatment with a chemotherapy drug. By thermal analyses, the cells were probed as micrometric reservoirs of bulk-like and confined water populations. Under this perspective we showed that the drug clearly alters the properties of the confined water. We have independently validated this idea by accessing the cellular water dynamics using inelastic neutron scattering. Finally, analysis of the quasi-elastic neutron scattering data allows us to hypothesize that, in this particular cell line, diffusion increases in the intracellular water in response to the action of the drug on the nanosecond timescale.

## Introduction

Finding new venues for studying and understanding cancer cells is one of the biggest challenges in current medical research. Specifically, predicting if a tumor is prone to progression or remission is an uppermost priority^1^. To this end, different approaches have been attempted to anticipate the behavior of cancer cells before and after starting a treatment. Accordingly, cancer cells have not only been investigated under biological perspectives, which usually focus on individual cellular components, but also from materials science and biomechanical points of view, which study the cells as whole systems. For example, from measurements of the rigidity of malignant cells in comparison with benign cells it has been reported that the first are significantly softer. Nonetheless, depending on the investigated cell lines, the opposite behavior has also been described. It is therefore evident that elastic properties alone are not completely reliable in describing the properties of cancer cells as materials. Another important point is that, with certainty, during tumor progression changes occur not only in macroscopic structure and viscoelasticity in the cells, but also in their internal microscopic dynamics. This is expected since alterations in cells morphology and packing naturally influence the pathways through which organelles, water, and other biomolecules can move^2^. Intriguingly, despite the consolidation of this idea and the fact that cells primarily contain water molecules, very few studies have focused on the properties of cellular water (water inside and outside the cells) *per se* and related these to the behavior of cancer cells.

**Figure 1:**
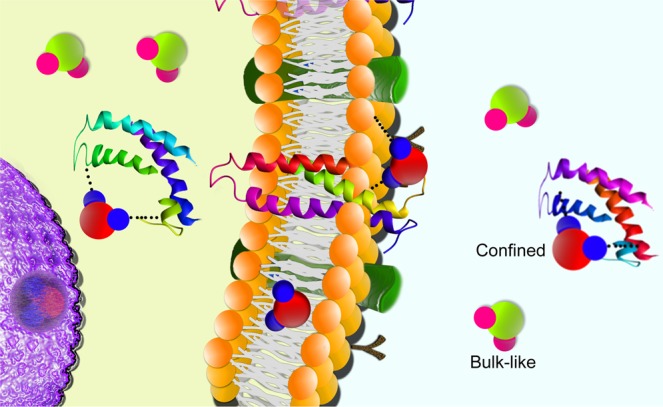
Representation of the co-existence of bulk-like and confined water populations inside and outside the cells. Weakly interacting water molecules show bulk-like behavior (represented in green and pink) and have a diffusion coefficient of about 3 × 10^−9^ m^2^/s. Water molecules, confined by folded proteins, cell membranes, organelles and others, exhibit restricted mobility (represented in red and blue).

Given the overcrowded nature of the cellular medium, deviations in overall properties of water may be related to single or distinct types of motions and this differentiation can be the key to explain how and why water dynamics change within cells with different prognoses^3^. These possible differences are caused by the distinct ways that water may interact within the complex cellular environment^4^. While weakly interacting water molecules show bulk-like behavior, diffusing at 37 °C with a diffusion coefficient of about 3 × 10^−9^ m^2^/s, water molecules confined by, for example, folded proteins, cell membranes and organelles, exhibit different properties since their mobility is restricted and their effective diffusion is therefore reduced^5^. As illustrated in Fig. 1, bulk-like (represented in green and pink) and confined (represented in red and blue) water populations co-exist inside and outside the cells. Additionally, the culture medium surrounding the cells contains enzymes that may also lead to the presence of confined water, thus bringing great complexity to the analysis of experimental data.

One of the main techniques applied in characterizing cellular water is diffusion-weighted magnetic resonance imaging (DW-MRI) that has been used in various studies to differentiate, for example, normal and cancerous tissues. However, while the apparent diffusion coefficient (ADC) derived from this technique is a promising tool to assess treatment response, some inherent limitations in DW-MRI interpretation still exist^6^. While DW-MRI has provided crucial information on the microscopic mobility of water^7^, cell water dynamics on sub-micrometric and sub-nanosecond scales have not yet been comprehensively studied. Additionally, understanding the nature of the motions (rotational or translational) of water molecules within cells is limited in DW-MRI.

In this report, we suggest to tackle this problem and overcome the limitations of DW-MRI with a pure material science perspective. We combine sophisticated neutron scattering experiments with traditional thermal analysis to investigate the behavior of the water confined in breast cancer cells (MCF-7), either not treated (NTC) or treated (TC) with the anti-cancer drug paclitaxel (PTX). As PTX is known to arrest cells in mitosis, we were able to characterize water dynamics within a widely used cell model in a specific phase of the life cycle.

First, by comparing the water’s melting profiles, as observed by differential scanning calorimetry (DSC), it was possible to establish that the disturbance of water hydrogen bond network increases from the NTC to the TC^8–11^. Then, using inelastic neutron spectroscopy (INS), a direct probe of atomic and molecular motions, we demonstrate that, under the experimental conditions used in this work, this disturbance is related to the manner in which confined water molecules are structurally organized and interact in the cellular environment. Finally, the contribution from the bulk-like water population to the change in the water dynamics in MCF-7 cells after treatment with PTX is depicted by the quasielastic neutron scattering (QENS) results. Particularly, these show that PTX increases diffusional mobility in the intracellular bulk-like water population.

## Results and Discussion

**Figure 2:**
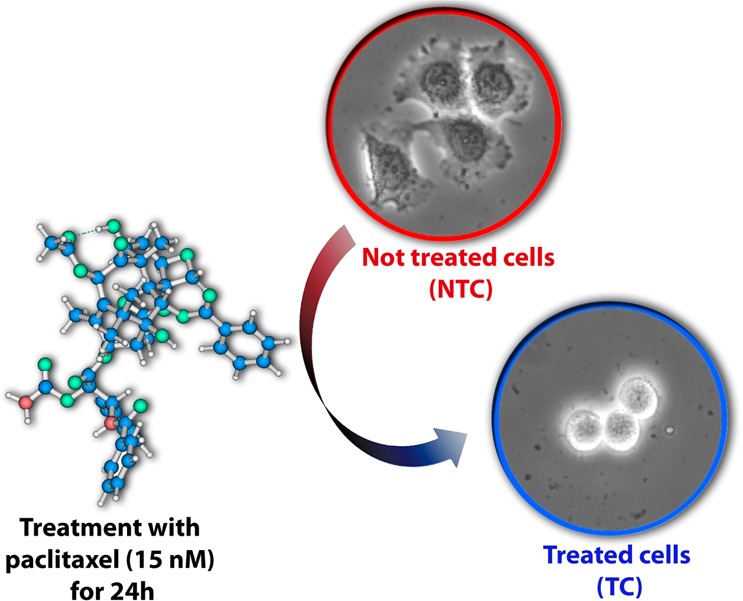
Representative optical microscopy images of breast cancer cells (MCF-7) not treated (NTC - red circle) and treated (TC - blue circle) with 15 nM of paclitaxel for 24 h. The rounded cell morphology observed in the TC is expected due to PTX’s action. The PTX molecule is shown in the left corner, where blue represents the carbon atoms, red represents nitrogen, cyan represents oxygen and the H atoms are shown in white.

All results were obtained in living cancer cells as confirmed by viability tests, which showed that around 70% of both treated cells (TC) and not treated cells (NTC) were still viable (i.e. alive) even after the neutron scattering experiments that were performed at room temperature for several hours. First, optical microscopy analysis was performed to observe morphological changes in human MCF-7 breast cancer cells due to PTX’s action. It is known that PTX’s action is driven by its interaction with microtubules and the consequent arrest of cells in mitosis^12^. As a consequence of this process, the rounded cell morphology observed in the TC is expected, as shown by the representative phase contrast microscopy images of the studied cells in Fig. 2.

**Figure 3:**
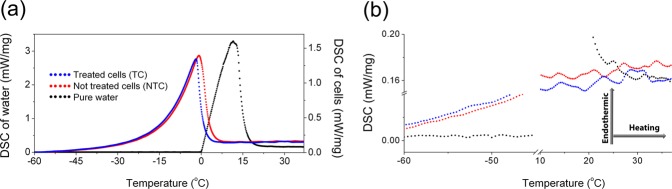
Differential scanning calorimetry (DSC) performed on bulk water (black circles) and on breast cancer cells (MCF-7) not treated (NTC - red circles) and treated (TC - blue circles) with 15 nM of paclitaxel for 24 h. (**a**) Respective melting peaks and (**b**) specific heats (baselines) before and after melting. The samples masses were approximately 25 mg.

In seeking to investigate how these morphological changes due to PTX action cause perturbations within cellular water, the cells were studied by means of differential scanning calorimetry (DSC) at temperatures around the water melting point. The results are given in Fig. 3(a,b), together with pure water (bulk) that was also analyzed for better comparison. The accessibility and fastness of this experiment allowed us to perform this analysis in duplicate using cell cultures independently prepared. Specifics are presented as Supplementary material.

Water, as a pure substance, has its melting point determined by the onset on the curve, while for water in the cells the melting point is determined by the peak position of the respective transitions^13^. As a result of a distribution of confined water populations, both TC and NTC show melting peaks with tails at the low temperature side. At the high temperature side, the cells exhibit melting rates similar to that observed for water, thus depicting the presence of a bulk-like water population. Regarding the NTC, the melting peak is centered at 0.8 °C, indicating a melting temperature close to that observed for water. Meanwhile, the melting peak of the TC is shifted to −1.8 °C and shows a 5% reduction of the area under the curve. This points to a reduction in the melting enthalpy, from 202 J/g for NTC to 192 J/g for TC (while for pure water we have determined the melting enthalpy as 367 J/g), thus showing that in the TC, the ice structure is somewhat less stable and suggesting a slightly higher content of confined water. After re-scaling the bulk water data to match the melting peaks from each cell’s data, we observe that bulk water represents around 60% of the signal in both cases. While not purely quantitative, this simple method provides an approximate amount of bulk water in the cells, which is within the range recently determined for distinct living cells^14^. Graphical representations of this analysis are also presented as Supplementary material.

Focusing now on the analysis of the baseline of each sample, as highlighted in Fig. 3(b), information about the specific heat of the NTC and TC can be obtained and compared before and after melting (solid and liquid phases, respectively). In the solid state, the TC likely have higher specific heat values, indicating an increase of phonon vibrations^15^ of the ice structure. Because of the very broad range of the melting transitions further confirmation of this assumption was needed. This was achieved by INS as described below. At biological temperatures, i.e. above melting, despite the oscillations in the data, the TC (blue line) shows lower specific heat, and thus stronger response to temperature changes.

**Figure 4:**
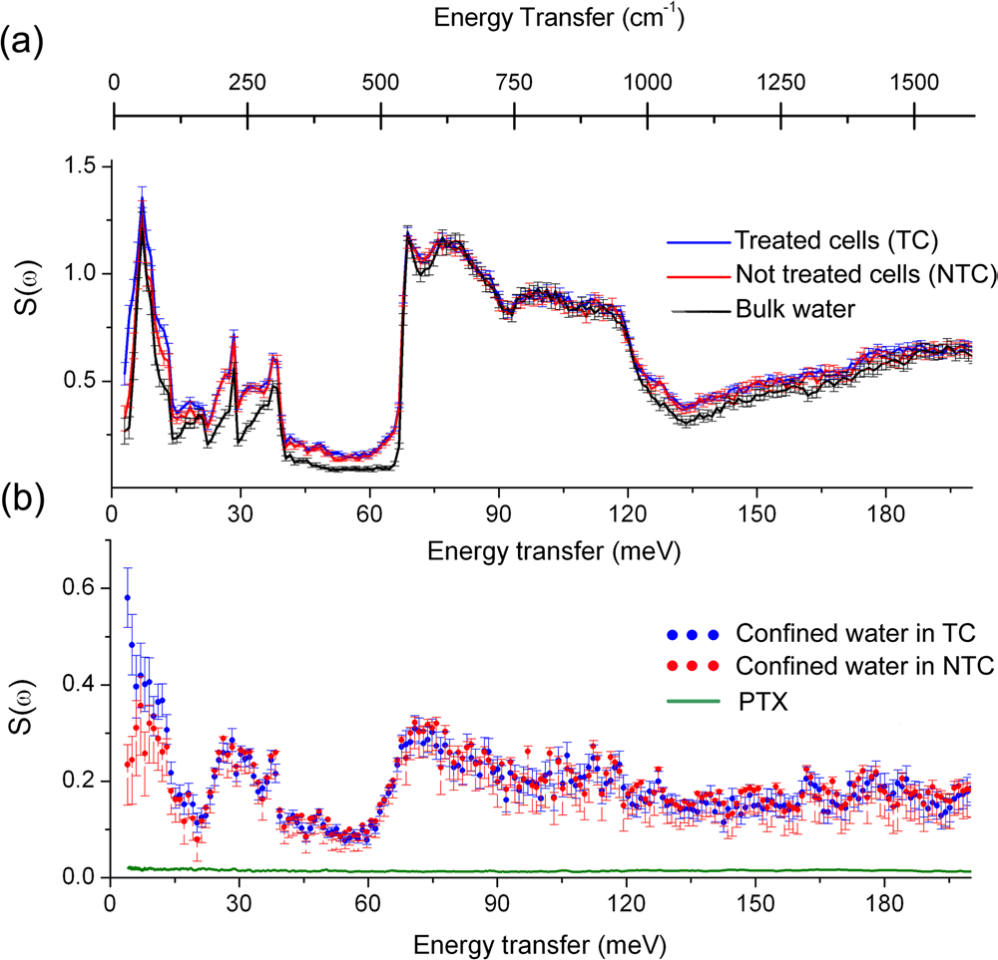
(**a**) Inelastic neutron scattering (INS) spectra for breast cancer cells (MCF-7) not treated (red) and treated (blue) with 15 nM of paclitaxel for 24 h and for bulk water (ice Ih form) (black). Data was collected on one sample of each cell group. The volumes of the samples were 2.5 mL and consisted of 3 × 10^7^ cells/mL. The presented results were obtained after 12 h of data collection for each sample allowing for an optimal fractional error bar as defined by Poisson statistics. (**b**) Contribution from the confined water of TC and NTC cells obtained after subtraction of bulk water contribution. Also in (**b**), the green curve shows INS data for PTX, which was scaled to reflect the drug concentration of 15 nM added to the TC. PTX spectrum is presented as Supplementary information within a scale that allows for observing the molecule vibrational modes together with their assignments.

To deepen our understanding on the changes in the cellular water behavior before melting, we turn to the INS experiments depicted in Fig. 4. INS data was obtained in a cumulative way for 12 hours at 10 K using the TOSCA spectrometer located at the ISIS Neutron and Muon Source, UK, on both types of cells, pure (bulk) water and pure PTX. Figure 4(a) presents the spectra for TC, NTC and bulk water. The latter has been re-scaled for better visualization, while the PTX spectrum, shown in Fig. 4(b), was scaled to reflect the drug concentration of 15 nM added to the TC. Particular regions of the cells’ spectra can be well described as follows. Contributions from amide groups from proteins and DNA^16^ are assigned to vibrations above 140 meV (1120 cm^−1^), while the spectral region between 50 and 130 meV (403 and 1048 cm^−1^) is mainly ascribed to the water librational modes, where the bulk-like population dominates the spectra. Between 20 and 45 meV (161 and 363 cm^−1^), the sharp peaks are assigned to stretching of water H-bonds, while the broad ones observed in the cell spectra around 32 and 25 meV (258 and 202 cm^−1^) result from torsional motion of CH_3_ groups in proteins and DNA^16^. Finally, the spectral region between 0 and 20 meV (161 cm^−1^) is attributed to lattice vibrations, i.e. phonon modes, of both water and proteins. This spectral region exhibits the clearest difference between NTC and TC spectra.

To gain further insight solely on the confined water contribution in the NTC and the TC, we have subtracted the bulk water contribution from both spectra, using the information obtained from the DSC analysis (60% of the water content in the cells have bulk-like behavior). For that, prior to data subtraction, we have re-scaled the INS data of bulk water between 50 and 130 meV (403 and 1048 cm^−1^), where all the contributions can be assigned to water molecules. Thus, the plots shown in Fig. 4(b) predominantly represent the contribution from confined water together with the remaining cell components. Following this approach, the subtle difference between the NTC and TC spectra at the lattice vibrations spectral region, 0 and 20 meV (161 cm^−1^), is further highlighted. This observation strengthens the calorimetric results that have already indicated that the action of the drug modifies the way in which water molecules are structurally arranged in MCF-7, validating the differences observed in the specific heat in the solid state. On the other hand, vibrations between 20 and 45 meV (161 and 363 cm^−1^), attributed to proteins and DNA molecules, are similar for both samples. Since the amplitude of vibrational bands are directly dependent on the concentration of vibrating species, this result shows that changes in confined water properties are not related to changes in proteins concentration within the MCF-7 samples.

Although the thermal analyses insights for the cells in the solid state can be explained based on changes of the lattice vibrations, as shown by INS, the same is not true for the cells above the melting point. In this case, following the description proposed by Bolmatov *et al*.^17^ to describe the thermal properties of liquid systems, it is expected that diffusional and rotational motions play an important role in the specific heat of the cells. Differences in the water dynamics above melting, already indicated by slight differences in the specific heat, are expected to be even more evident in the QENS data obtained at ambient temperature using the BASIS spectrometer located at the Spallation Neutron Source, Oak Ridge National Laboratory, USA. In these experiments, the samples were also measured for approximately 12 h for reduction of fractional error and optimization of signal-to-noise ratio. Here it is worth to mention that data collected during the first minutes were compared to the cumulative data and no changes neither in the INS nor in the QENS spectra were observed. This demonstrates the integrity of the samples as well as the reproducibility of the data.

In a QENS spectrum, dynamic components typically manifest themselves as Lorentzian curves, whose line-widths, Γ, behavior as a function of the scattering wave-vector, *Q*, allows differentiating diffusive and localized motions. While rotation of molecules do not show any clear Q-dependence in the QENS signal width, the same is not true for translational motions^18^. Also, by representing the QENS as the imaginary part of the susceptibility^19,20^:

1 $$\chi ^{\prime\prime} (Q,E)\propto \frac{S(Q,E)}{{n}_{B}(T,E)}=S(Q,E)\ast [exp(\frac{E}{kT})-1]$$

where *n*_*B*_(*T*, *E*) is the temperature Bose factor, different relaxation processes can be clearly separated; changes in peaks position, shape and/or intensity are good indications for Q-dependent processes, i.e. water diffusion.

Because the motions of purely bulk water molecules are usually too broad (fast) to be discernable from the background due to the instrumental design of BASIS, any bulk-like behavior observed in this descriptive study is likely attributable to water inside the cells, rather than extracellular water, whose dynamic properties are expected to be much closer to pure bulk behavior^20^.

**Figure 5:**
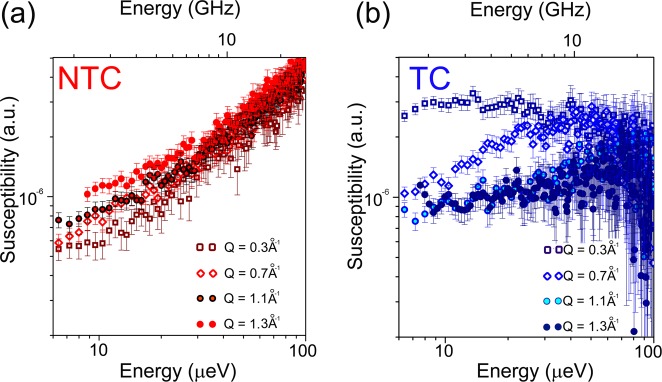
Dynamic susceptibilities obtained from QENS data for not treated breast cancer cells (MCF-7), NTC (**a**) and breast cancer cells treated with 15 nM of paclitaxel for 24 h, TC (**b**). The presented results were obtained after approximately 12 h of data collection for each sample allowing for an optimal fractional error bar as defined by Poisson statistics. Data was collected on one sample of each cell group. The volumes of the samples were 2.5 mL and consisted of 3 × 10^7^ cells/mL.

Figure 5(a,b) show respectively the imaginary parts of the susceptibility for selected *Q*-values for the NTC and TC. While for the NTC relatively little Q-dependence is detected, the opposite is observed for the TC. After being treated with PTX, the MCF-7 cells present a Q-dependent relaxation, centered around 10 μeV (2.4 GHz) at Q = 0.3 Å^−1^ and is gradually shifted to higher frequencies as Q increases. For these cells, we can therefore infer the presence of non-localized (diffusive) motions on the time and length scales assessed by the instrument.

**Figure 6:**
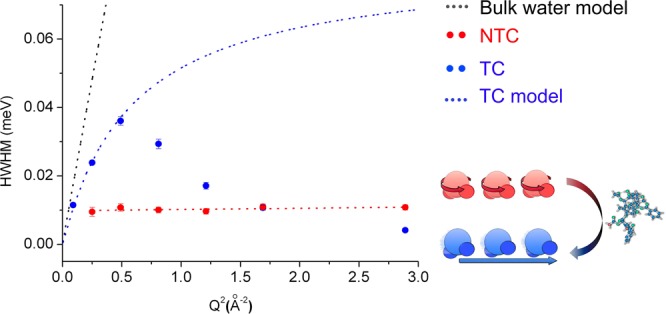
Evolution of QENS broadening (half width at half maximum – HWHM), determined from fits (see Experimental section and supplementary material) and attributed to the water molecule motion in breast cancer cells (MCF-7), as a function of Q^2^. The symbols represent the values obtained when the HWHM was allowed to vary independently at each Q-values, while the error bars represent the standard deviation. Note that some of the error bars are within the dots. The black dotted line represents a theoretical curve for bulk water obtained from the jump diffusion model, Equation (2). The same model was used to obtain the blue dotted curve, which describes the behavior of the TC. The red dotted line represents a linear fit that describes the HWHM behavior in the NTC. The data collected at Q^2^ = 2.25 Å^−2^ were not used due to the presence of a Bragg reflection. In the bottom right corner, the scheme illustrates the appearance of translational motions in the cells after treatment with PTX.

The presence of diffusive motions in the TC is further confirmed and quantitatively discussed by the analysis of the evolution of the QENS broadenings (half width at half-maximum, HWHM) *vs* Q^2^ presented in Fig. 6. Here, the HWHM values were obtained by performing a nonlinear least square fitting to the QENS signal at different Q-values. A single Lorentzian function, convolved with the instrumental resolution was used as input model together with a baseline correction that accounted for the background. The χ^2^ (chi-squared) test was used as the statistical analysis tool for goodness-of-fit. The results are presented as Supplementary material together with the graphical representations of the fits. For comparison purposes, Fig. 6 also shows a theoretical curve depicting the expected behavior for purely bulk water at room temperature (black dotted line), obtained by considering the jump diffusion model, described by the expression below^21^:

2 $$HWHM(Q)=\frac{D{Q}^{2}}{1+D{Q}^{2}{\tau }_{0}}$$

where D refers to the diffusion coefficient and τ_0_ is the residence time. For bulk water we have used D = 2.9 × 10^−9^m^2^/s and τ_0_ = 1ps^22^.

For TC, the QENS broadening presents a clear Q-dependence below 0.7 Å^−1^, confirming the presence of non-localized, long-range translational motions. Above this value, the motions become too fast for the instrumental range of accessible energy transfers and fitted values of the measured signals gradually decrease. On the other hand, for the NTC, the HWHM is Q-independent, indicating spatially localized (rotation motions) and short-range relaxations. These observations quantify the conclusions drawn from the model-independent susceptibility data, thus offering clear evidence for a less spatially constrained mobility of the intracellular water molecules in the TC when compared to that in the NTC. The proposed change in dynamics behavior before (red) and after treatment (blue) with PTX is illustrated in the bottom right corner of Fig. 6.

Sequentially, the curve obtained for the expected behavior of bulk water via Equation (2) was adjusted to the data from the TC below 0.7 Å^−1^ by a nonlinear fitting procedure. Hence, the curve describing the water dynamics in the TC was obtained with D_TC_ = 2.21 ± 0.11 × 10^−9^ m^2^/s and τ_0-TC_ = 9.09 ± 0.62 ps. While the diffusion coefficient, D_TC_, is comparable to the one expected for bulk water, the significantly higher residence time shows that the H-bond network in this water population is considerably disturbed. The same is true for the NTC, where a rotational relaxation time of about 67 ± 3 ps can be extracted (${\tau }_{{rotation}}\,\approx \,2\hslash /{{\rm{\Gamma }}}_{r}$^23^) by a linear fitting as presented in Fig. 6 by the red dotted line. In both cases, the error bar depicts the standard deviation of the HWHM value determined by the fit. The comparison clearly demonstrates that the water structural network in the TC differs from the one present in the NTC and that there is higher mobility freedom for the water in the TC.

## Conclusions

Our data show that neutron spectroscopy combined with thermal analysis provides new insight on cellular water motions on the sub-nanosecond time scale within living MCF-7 cells on the sub-micrometric length scale. Thermal analysis clearly reveals that after interaction with the anti-tumor drug PTX, the cancer cells (TC) present thermal behavior even further away from that of bulk water than not treated cells (NTC). Specifically, the TC present reduced melting enthalpy and distinct specific heat in the solid and liquid states. In fact, the lower specific heat at room temperature for TC, and consequently higher susceptibility to temperature variations under heat is of great interest and must be further investigated for different cell lines. While the effects of hyperthermia treatments in improving the drug’s action are well known^24^, the inverse, i.e. the drug effect on hyperthermia’s performance, has not yet been clearly investigated based on physical characterization techniques.

Differences in the dynamics of cellular water within the investigated breast cancer cells line before and after treatment with PTX were further confirmed by neutron spectroscopy. In this case, the data show amplification of collective motions from confined water population in the TC, which is, interestingly, rather independent on the concentration of proteins in the sample. Additionally, the QENS data reveal that intracellular bulk-like water population also contributes to the changes in cellular water dynamics after the action of the drug. Based on a theoretical hypothesis, it is demonstrated that in the TC the cellular water has translational diffusional mobility on the probed time and length-scales, which is not observed for the NTC. For the latter, the observed behavior considerably deviates from the theoretical expectation of jump diffusion. Therefore, the findings of this descriptive work concur with the hypothesis that in the cultured MCF-7 cells cellular water dynamics plays an active role in cell cycle and in cellular response to external stimuli. Certainly, this result is a first step in using solid state techniques, i.e. neutron scattering and thermal analyses, in describing how water mobility in cancer cells differs to the water mobility observed in healthy and/or treated cells. Future investigations with different cell lines and distinct chemotherapy agents must be undertaken to fully demonstrate that the ongoing advances in neutron scattering and its combination with traditional materials science techniques can indeed provide the opportunity to revisit the role played by water in living organisms.

## Experimental Section

### Cell culture

Human breast cancer cells (MCF-7) were propagated at 37 °C in Dulbecco’s Modified Eagles Medium (DMEM) supplemented with 10% fetal calf serum, 2 mM glutamine, 5000 IU/mL penicillin and streptomycin in a humidified atmosphere containing 5% CO_2_. Once the cells were confluent they were washed with phosphate buffered saline (PBS: 10 mM Na_2_HPO_4_, 1.8 mM KH_2_PO_4_, 137 mM NaCl, 3 mM KCl, pH 7.4), and incubated in DMEM without serum. Next, half of the cells were treated with paclitaxel (PTX) (LC Laboratories) with a final concentration of 15 nM for 24 hours. The PTX dose was defined based on its IC50 value^25^ and the treatment period was adopted to ensure that most cells were arrested in mitosis (displaying the round morphology). Both the cells treated (TC) and not treated (NTC) with PTX were subsequently dislodged using trypsin and transferred to DMEM containing serum and washed with PBS by centrifugation (1000 g, 10 min). The cells were then re-suspended in 4 mL DMEM with 10% DMSO but without serum at 3 × 10^7^ cells/mL. For the thermal analyses, 25 mg of TC and NTC were reserved while for inelastic neutron scattering (INS) experiments, 2.5 mL of each group was mounted in flat aluminum sample holders. In both cases, the cells were immediately frozen and stored at −80 °C until use.

The procedure described above was performed twice. First, cultures of NTC and TC were prepared for the experiments performed at TOSCA. Then, a few months later, new cultures of NTC and TC were prepared for the experiments performed at BASIS. Representative populations of all cell cultures were investigated by thermal analysis (data presented as Supplementary material).

### Cell viability

Cell viability was tested by staining with Trypan Blue (Sigma-Aldrich) after concluding all the neutron scattering experiments, which were performed at room temperature for several hours. Around 70% of both TC and NTC were found to be viable.

### Microscopy

In order to perform morphological analysis, MCF-7 cells were seeded in 6 cm plastic dishes and then either treated or not treated with PTX as above. After 24 hours the cells were washed in PBS and fixed with formaldehyde (3% in PBS) for 15 minutes. After three washes with PBS, phase contrast images were acquired using a Leica CTR6000 microscope fitted with a 20X objective and a Leica DFC450C camera.

### Differential scanning calorimetry (DSC)

The thermal behavior of TC and NTC was evaluated by differential scanning calorimetry (DSC) using a Netzsch DSC 214 Polyma calorimeter. For such an experiment, the suspensions containing TC and NTC cells (3 × 10^7^ cells/mL) were taken out from storage at −80 °C and 25 mg of each was immediately mounted in sealed aluminum crucibles and placed in a nitrogen atmosphere purged at 40 ml/min. The same temperature calibration file, establishing the relationship between the temperature *T*_meas_ indicated by the instrument and the true temperature *T*_tr_: *T*_tr_ = *T*_meas_ + ∆*T*_corr_(*T*_meas_), was used for all measurements. The first data were collected on the cells prepared for the experiments conducted at TOSCA. In this case, no enthalpy calibration was used and the crucibles were placed into the calorimeter and rapidly cooled to −100 °C (20 °C/min). In a second experiment, with the cells prepared for the experiment at BASIS, a calibration enthalpy file was also used. Additionally, a protocol for system stabilization was followed for initial cooling of the samples to −100 °C. After the initial cooling, NTC and TC were subjected to a DSC cycle as follows: 1) heat to 37 °C (5 °C/min), 2) isothermal at 37 °C for 60 min, 3) cooling to −96 °C (5 °C/min) and 4) heat to 37 °C (5 °C/min). The data for the full cycles are presented as Supplementary Material. The data from the step (1) of the cycle, heat to 37 °C (5 °C/min), collected with the enthalpy calibration are presented in Fig. 3. Such a figure presents also data collected with a sample of 25 mg of distilled water subjected to the step (1) of the DSC cycle. Pure PTX powder (30 mg) was also analyzed without enthalpy calibration under heat from −96 °C to 220 °C (5 °C/min). This result is presented as Supplementary Material. Prior to the experiments an empty crucible was used as reference material.

### Neutron spectroscopy, uncertainties in the data and in the theoretical model fitting

The vibrational dynamics of TC and NTC were investigated by means of inelastic neutron scattering (INS). The experiments were conducted at the TOSCA neutron spectrometer at ISIS, UK^26^. As described before, similar volumes (2.5 mL) of TC and NTC suspensions were placed in flat thin-walled aluminum holders (sample thickness 1 mm) and each was measured at a temperature of 10 K. As neutron scattering experiments are inherently counting experiments, the uncertainty of the data is defined by Poisson Statistics, $\delta (N)=\sqrt{N}$, where N is the number of counts in a given frequency bin. Hence, the fractional error is $\frac{\delta (N)}{N}=\frac{1}{\sqrt{N}}$. In order to obtain an improved fractional error and by extension a satisfactory signal-to-noise ratio, the samples were measured for approximately 12 h. Subsequently, 0.88 g of PTX powder and 2.5 mL of pure distilled water were also recorded separately under the same experimental conditions described for the cancer cells.

Quasi-elastic neutron scattering (QENS) measurements were performed at the Backscattering Spectrometer BASIS, at the ORNL’s Spallation Neutron Source (SNS), USA^27^. The standard operation mode of BASIS allows for data collection within the energy range of +/−100 μeV (~6 ps), with a Q-averaged resolution of 3.5 μeV (FWHM) and a Q range of 0.2–2.0 Å^−1^. For these experiments, the cancer cells were mounted in aluminum plates by following the same procedure as the one described for INS experiments and measured at ambient temperature. Similarly to the description of the INS experiments, an improved fractional error was achieved by measuring the samples for approximately 12 h. After collecting the data, the QENS signal of each sample was analyzed at different Q-values and values for the Half Width at Half Maximum (HWHM) were obtained by using the software DAVE^28^ that uses the Levenberg-Marquardt nonlinear least squares with numeric differentiation to minimize the total sum of squares (TSOS) between S(Q,ω)_obs_ and S(Q,ω)_calc_. A single Lorentzian function (i.e. a mono exponential decay) convolved with the instrumental resolution was used as input model together with a baseline correction that accounted for the background and the χ^2^ (chi-squared) was used to evaluate the goodness of the fits. The HWHM values extracted from the cancer cells were compared with the theoretical expectation for bulk water.

## Supplementary information

Supplementary material
