# Supplementary material for: Water dynamics in MCF-7 breast cancer cells: a neutron scattering descriptive study
Source: Sci Rep. 2019 Jun 18;9:8704. doi: 10.1038/s41598-019-45056-8 (PMC6581907; doi:10.1038/s41598-019-45056-8)
Supplement: Supplementary file 1 — Supplementary material [file 41598_2019_45056_MOESM1_ESM.docx]

**Supplementary material**

**Water dynamics in MCF-7 breast cancer cells: a neutron scattering descriptive study**

Murillo L. Martins^1,2^, Alexander B. Dinitzen^3^, Eugene Mamontov^4^, Svemir Rudić^5^, José E. M. Pereira^1^, Rasmus Hartmann-Petersen^3^, Kenneth W. Herwig^4^ and Heloisa N. Bordallo^1,6^

*1 Niels Bohr Institute, University of Copenhagen, DK-2100 Copenhagen, Denmark*

*2 System and Production Engineering Graduate Program, Pontifical Catholic University of Goias, 74605-010 Goiania, Brazil*

*3 Department of Biology, University of Copenhagen, DK-2200 Copenhagen, Denmark*

*4 Neutron Scattering Division,Neutron Sciences Directorate, Oak Ridge National Laboratory, Oak Ridge, TN 37831,* *United States*

*5 ISIS Facility, Rutherford Appleton Laboratory, Chilton, Didcot OX11 OQX, UK*

*6 European Spallation Source, PO Box 176, SE-221 00 Lund, Sweden*


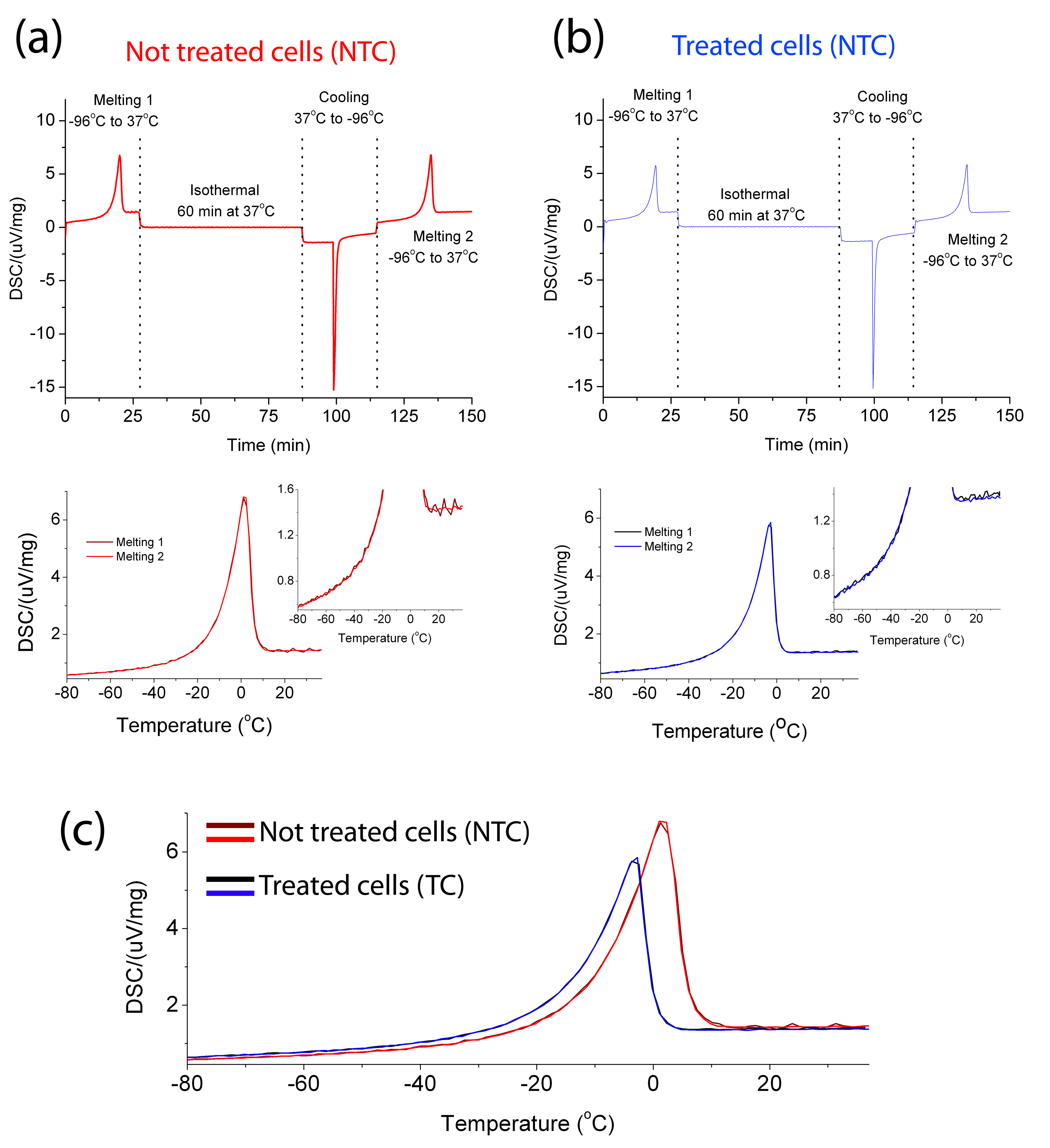


Figure SI1. Differential scanning calorimetry (DSC) performed on breast cancer cells (MCF-7) not treated (NTC) (a) and treated (TC) (b) with 15 nM of paclitaxel for 24h. The figures in the top show the full cycle to which the cells were subjected: first, the rapidly cooled to ~-96^o^C, and the frozen cells were heated from -96ºC to 37ºC (Melting 1), then an isothermal analysis was conducted for 60 min at 37ºC, the cells were cooled again to -96ºC and heated to 37ºC leading to Melting 2. The figures in the bottom show the comparisons between Melting 1 and 2 for both TC and NTC samples. In both cases, there is only difference between the data in the noise at the baseline, as highlighted by the inset. In (c) a comparison between the melting peaks of TC and NTC is presented. Enthalpy calibration was not enabled during the measurements leading to the μV/mg unit for the “heat exchange” values. The cells used in these experiments belong to the same batch than the ones used in the experiments performed on TOSCA. They were cultured independently to the cells used in the experiments performed at BASIS. The raw data are available in ref [1].


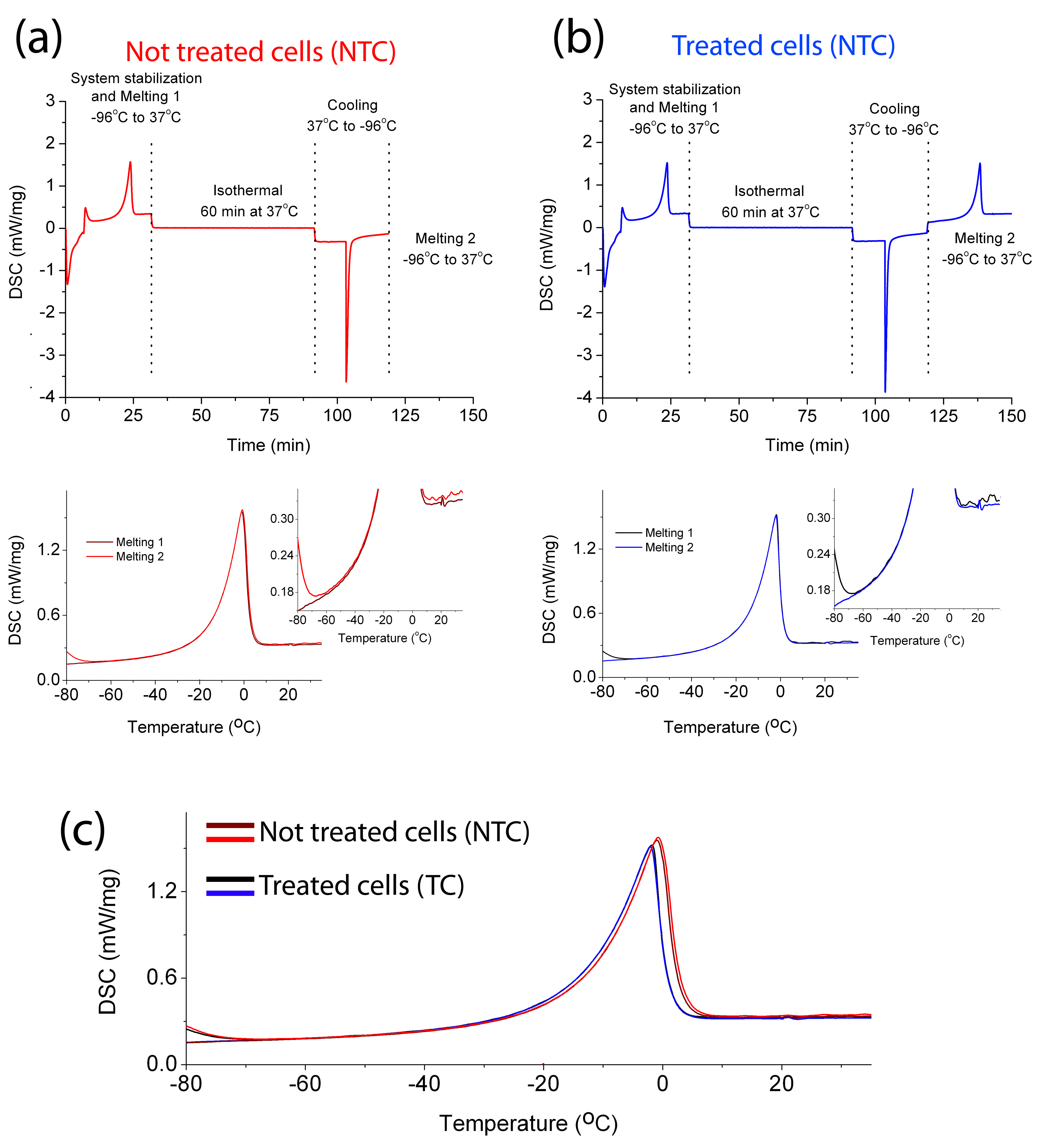


Figure SI2. Differential scanning calorimetry (DSC) performed on breast cancer cells (MCF-7) not treated (NTC) (a) and treated (TC) (b) with 15 nM of paclitaxel for 24h. The figures in the top show the full cycle to which the cells were subjected. First, the system was cooled following the protocol for system stabilization presented in Figure SI3. Subsequently, the frozen cells were heated from -96ºC to 37ºC (Melting 1), and an isothermal analysis was conducted for 60 min at 37ºC. Finally, the cells were cooled again to -96ºC and heated to 37ºC leading to Melting 2, which matches the melting detected in the first cycle, i.e. Melting 1. The figures in the bottom show the comparisons between Melting 1 and 2 for both TC and NTC samples. In both cases, there is only difference between the data in the noise at the baseline, as highlighted by the inset. In (c) a comparison between the melting peaks of TC and NTC is presented. The cells used in these experiments belong to the same batch than the ones used in the experiments performed on BASIS. They were cultured independently to the cells used in the experiments performed at TOSCA. . The raw data are available in ref [1].


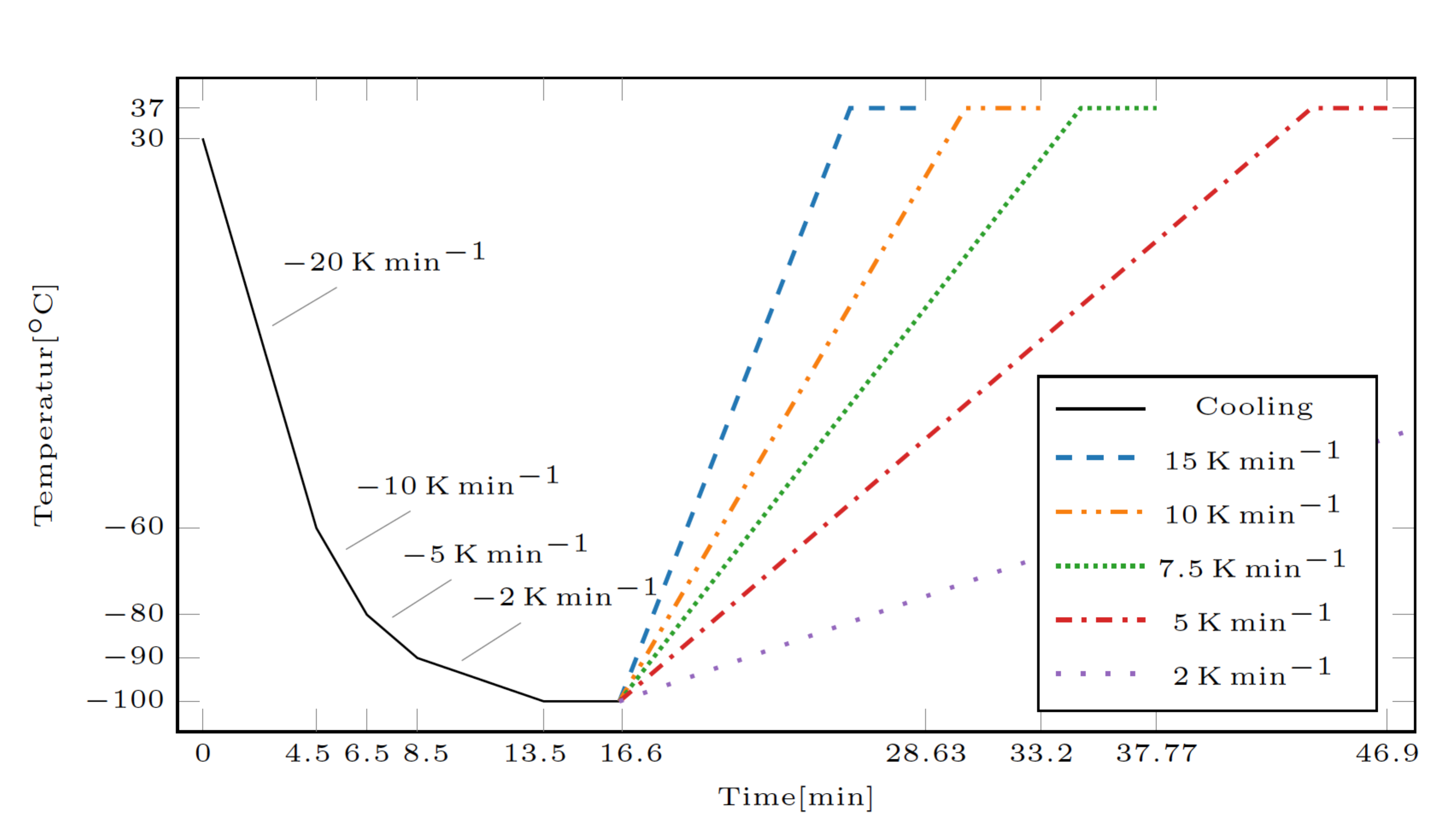


Figure SI3. Protocol for stabilization of DSC instrument based on cooling the system in divided steps. This protocol was used to obtain the data depicted in Figure SI2 for the cells measured in BASIS as well as for bulk water (Figure SI5).

**
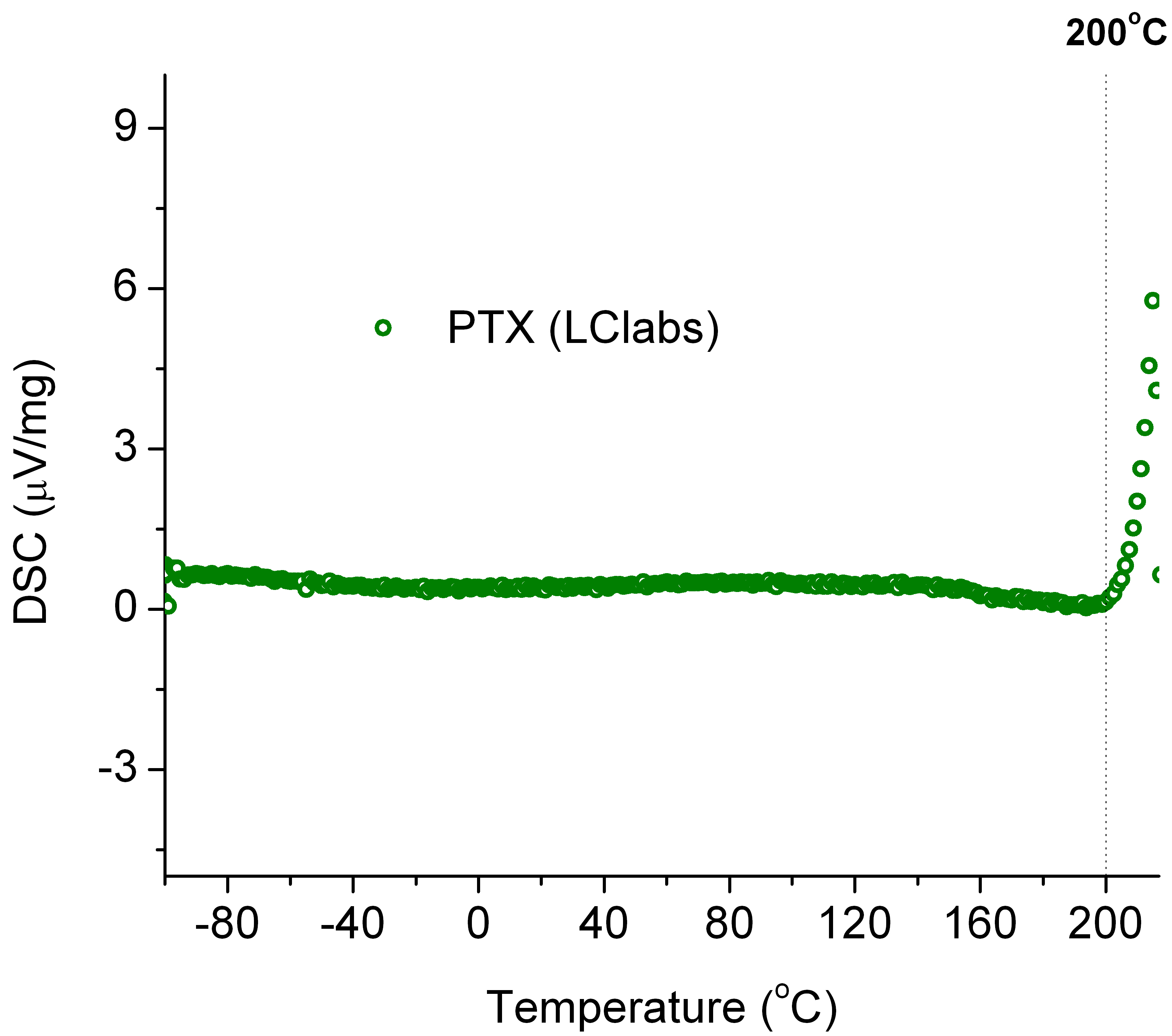
**

Figure SI4. Differential scanning calorimetry (DSC) performed on the paclitaxel (PTX) sample (LCLabs). The sample was heated from -96^o^C to 220^o^C. The enthalpy calibration was not enabled during the measurements leading to the μV/mg unit for the “heat exchange” values.

**
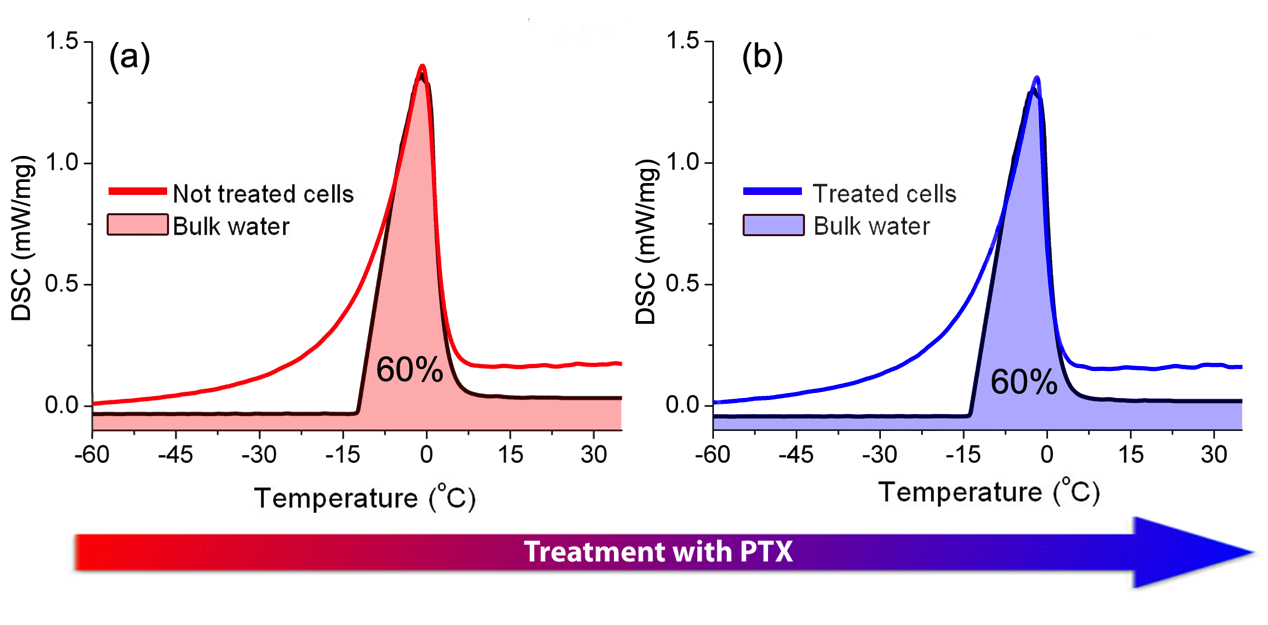
**

Figure SI5. Estimation of bulk water content based on the differential scanning calorimetry analysis. In (a) data from not treated breast cancer cells (MCF-7) (NTC) is presented while (b) shows the data from cells treated with paclitaxel (TC). The bulk water data was re-scaled so its melting peak could match the melting peak of the cells data. In both cases, the area in the melting peak covered by the bulk water data is around 60%. The cells used in this experiment belong to the same batch than the ones used in the experiments performed on BASIS.


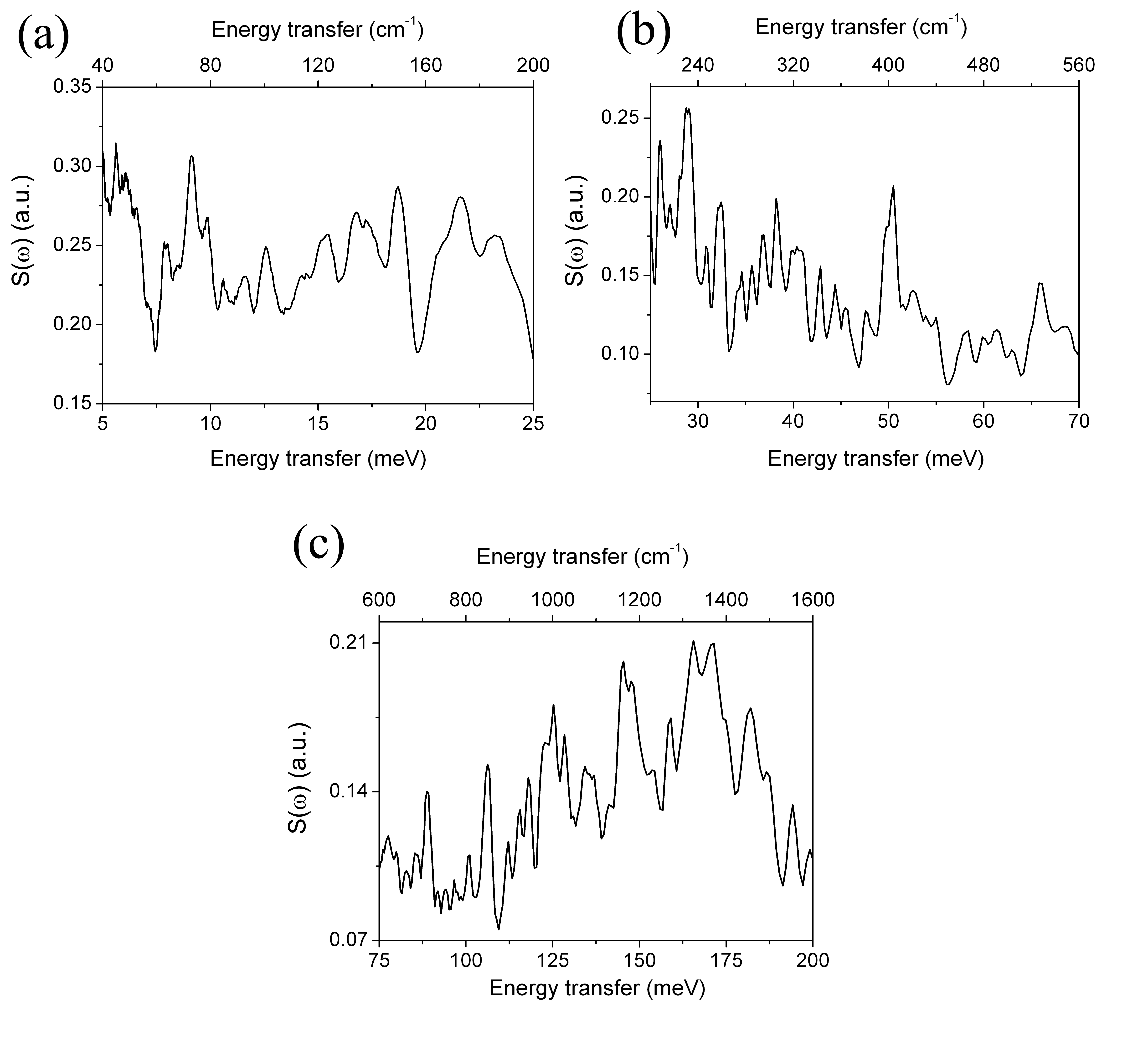
Figure SI6. Inelastic neutron scattering (INS) spectrum for paclitaxel. For better visualization, the spectrum is presented in three separated spectral ranges in (a), (b), and (c). The data has been collected at the TOSCA neutron spectrometer at ISIS, UK. Flat representations of the paclitaxel´s and similar molecules can be found in Ref [2].

Table SI1. Most relevant contributions for the vibrational modes observed in the inelastic neutron scattering spectrum of paclitaxel. AR denotes the Aromatic Rings. The numbering of the carbons follows the definition of Ref [2]. The main contributions were obtained from DFT calculations available in Ref. [3].

| **Frequency** | | **Main contributions** |
| --- | --- | --- |
| **meV** | **cm^-1^** |  |
| 6 | 45 | AR1 and oxetane ring |
| 8 | 68 | Acetyl linked to C10 and AR1 |
| 9 | 74 | Acetyl groups, AR1 and oxetane ring |
| 11 | 85 | Oxetane ring, methyl groups in C16, C17 and C19 and C6 |
| 12 | 96 | Acetyl linked to C4, methyl groups in C16, C17, C18 and C19, AR1 and terpene ring |
| 14 | 109 | Terpene ring, AR3 and Acetyl linked to C4 |
| 16 | 131 | Terpene ring, oxetane ring and methyl in C17 |
| 17 | 140 | Side chain in C13 and methyl groups in C16 and C17 |
| 19 | 152 | AR2, AR3, methyl groups in C16 and C17 |
| 22 | 177 | Terpene ring, |
| 23 | 184 | Methyl groups in C17 and C18, AR3, Acetyl linked to C4 and terpene ring |
| 25 | 205 | Terpene ring and methyl groups in C17 and C18 |
| 26 | 212 | Methyl groups in C16, C17 and C19 and Acetyl linked to C10 |
| 27 | 222 | Methyl groups in C16, C17 and C19 and terpene ring |
| 28 | 226 | Methyl in C19 and oxetane ring |
| 31 | 253 | Side chain in C13 and methyl in C18 |
| 34 | 273 | Methyl groups in C16 and C19 and Acetyl linked to C4 |
| 37 | 302 | AR1, Acetyl linked to C4 and methyl groups in C18 and C19 |
| 41 | 335 | Methyl in C16, OH bonded to C7 and side chain in C13 |
| 48 | 387 | OH bonded to C7, side chain in C13 and oxetane ring |
| 50 | 399 | OH bonded to C1, methyl group in C15 and terpene ring |
| 51 | 411 | OH bonded to C7, oxetane ring and methyl groups in C18 and C19 |
| 65 | 527 | NH and OH groups in side chain linked to C13 and acetyl linked to C10 |
| 89 | 717 | AR1, oxetane ring, terpene ring, methyl group in C15 |
| 106 | 852 | AR2 |
| 117 | 943 | AR3, methyl groups in C15 and C8 and acetyl linked to C10 |
| 133 | 1070 | Acetyl linked to C4 |
| 143 | 1158 | Terpene ring, oxetane ting and methyl in C15 |
| 159 | 1281 | AR1, acetyl groups |
| 164 | 1323 | AR2, C1`and C2` |
| 169 | 1360 | AR2, C1`and C2` |
| 180 | 1452 | Methyl groups in C16, C17 and C19 |
| 192 | 1552 | Methyl groups in C16, C17 and C19 |

** AR1 stands for the aromatic ring in the side chain linked to C2*

** AR2 stands for the aromatic ring linked to C3`*

**AR3 stands for the aromatic ring linked to C5`*


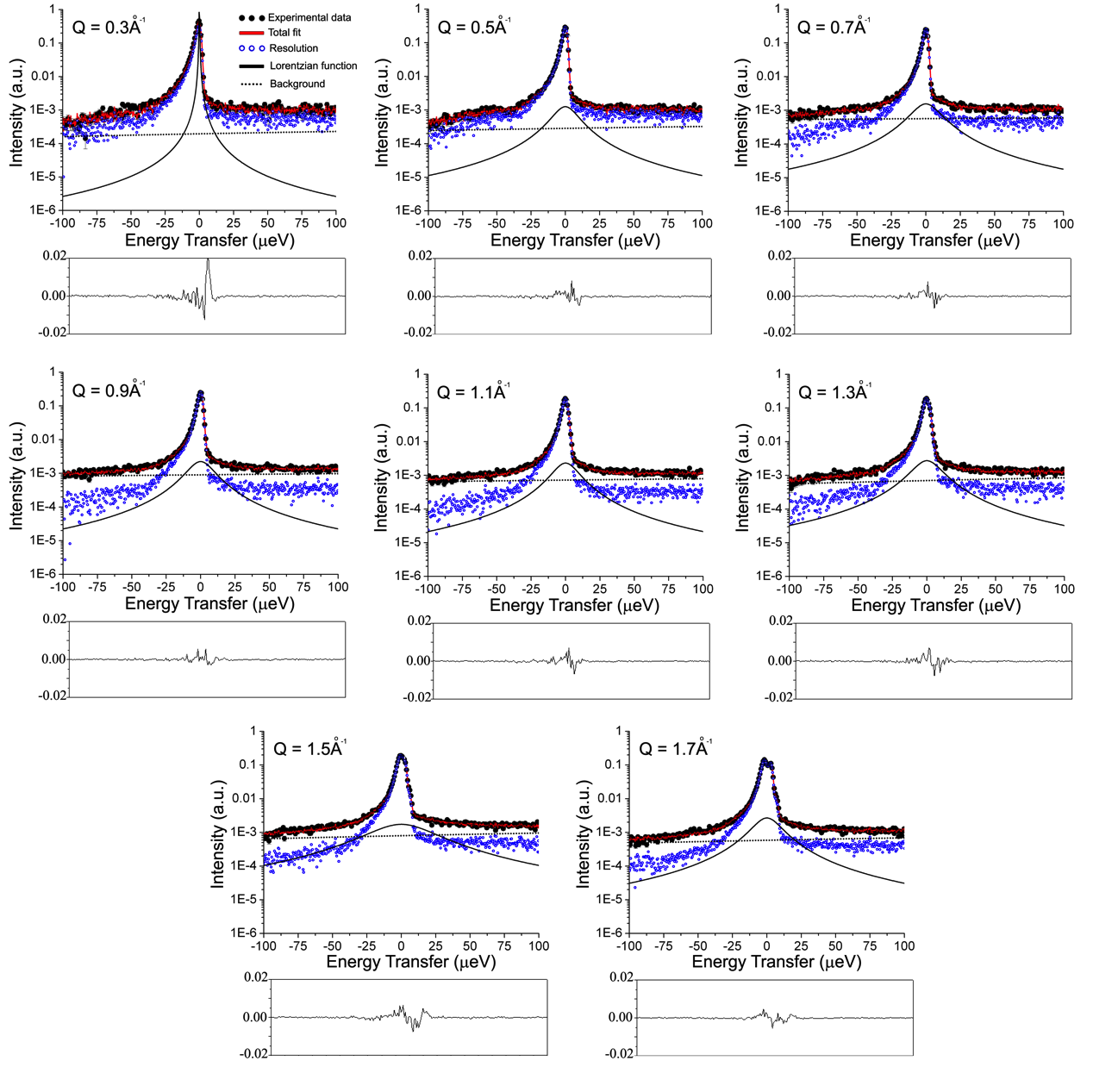


Figure SI7. Quasi-elastic neutron scattering data collected at 310K at different values of Q of breast cancer cells (MCF-7) not treated with paclitaxel (NTC). The data were fitted by single Lorentzian functions (black line) and a background (back dotted line) convolved with a resolution function. The latter was obtained by the convolution of a delta function and the data collected at 10K and is presented in blue. The total fit curves are presented as red lines. The boxes in the bottom depict the difference curves between experimental data and the fit curves.


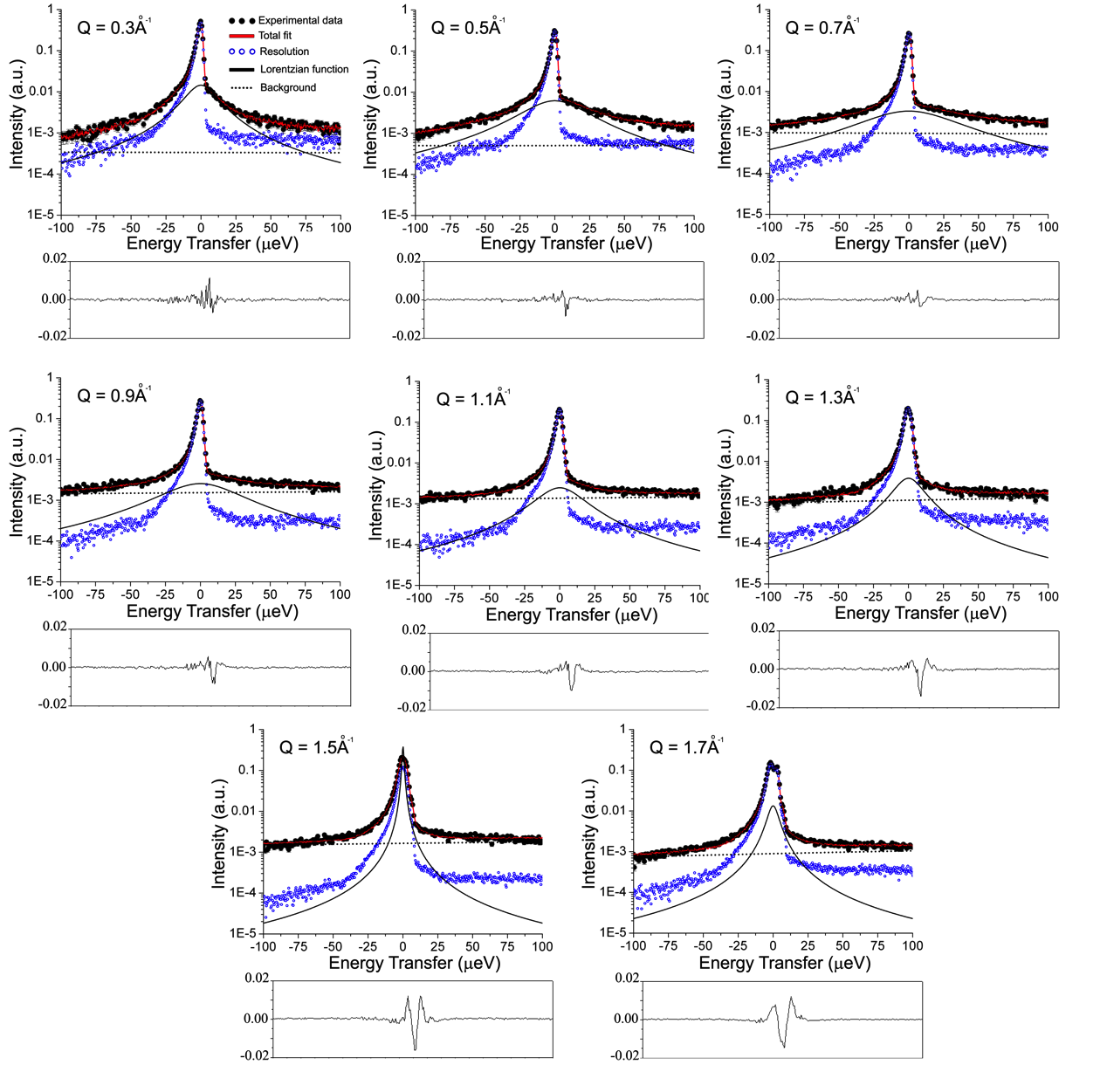


Figure SI8. Quasi-elastic neutron scattering data collected at 310K at different values of Q of breast cancer cells (MCF-7) treated with paclitaxel (TC). The data were fitted by single Lorentzian functions (black line) and a background (back dotted line) convolved with a resolution function. The latter was obtained by the convolution of a delta function and the data collected at 10K and is presented in blue. The total fit curves are presented as red lines. The boxes in the bottom depict the difference curves between experimental data and the fit curves.

Table SI2. χ^2^ (chi-squared) values used to evaluate the goodness of the fits performed by a nonlinear least square method to obtain the Half Width at the Half Maximum (HWHM) of the QENS signals at different Q^2^-values.

| **Sample** | **Q^2^-value (Å^-1^)** | **χ^2^** |  | **Sample** | **Q-value (Å^-1^)** | **χ^2^** |
| --- | --- | --- | --- | --- | --- | --- |
| NTC | 0.09 | 2.376 |  | TC | 0.09 | 1.305 |
|  | 0.25 | 2.493 |  |  | 0.25 | 1.560 |
|  | 0.49 | 1.880 |  |  | 0.49 | 1.279 |
|  | 0.81 | 1.648 |  |  | 0.81 | 1.441 |
|  | 1.21 | 1.682 |  |  | 1.21 | 1.606 |
|  | 1.69 | 1.616 |  |  | 1.69 | 1.854 |
|  | 2.25 | 1.904 |  |  | 2.25 | 2.326 |
|  | 2.89 | 2.229 |  |  | 2.89 | 3.831 |

[1] Bordallo, H. N., & Martins, M. L. (2019). Differential scanning calorimetry (DSC) data for breast cancer cells. ESS. ([doi.org/10.17199/NXMV03.DSC0001](https://doi.org/10.17199/NXMV03.DSC0001))

[2] Mastropaolo D, Camerman A, Luo Y, Brayer GD, Camerman N. Crystal and molecular structure of paclitaxel (taxol). Proc. Natl. Acad. Sci. USA 1995; 92; 6920 - 6924.

[3] Martins, M. L., *et al*. Restricted mobility of specific functional groups reduces anti-cancer drug activity in healthy cells. Scientific Reports, **6**, 22478, 2016.
